# The effects of radiation on angiogenesis

**DOI:** 10.1186/2045-824X-5-19

**Published:** 2013-10-26

**Authors:** Peter Grabham, Preety Sharma

**Affiliations:** 1 Center for Radiological research, Columbia University, VC 11-243, 630 West 168th street, New York, NY 10032, USA

## Abstract

The average human body contains tens of thousands of miles of vessels that permeate every tissue down to the microscopic level. This makes the human vasculature a prime target for an agent like radiation that originates from a source and passes through the body. Exposure to radiation released during nuclear accidents and explosions, or during cancer radiotherapy, is well known to cause vascular pathologies because of the ionizing effects of electromagnetic radiations (photons) such as gamma rays. There is however, another type of less well-known radiation – charged ion particles, and these atoms stripped of electrons, have different physical properties to the photons of electromagnetic radiation. They are either found in space or created on earth by particle collider facilities, and are of significant recent interest due to their enhanced effectiveness and increasing use in cancer radiotherapy, as well as a health risk to the growing number of people spending time in the space environment. Although there is to date, relatively few studies on the effects of charged particles on the vascular system, a very different picture of the biological effects of these particles compared to photons is beginning to emerge. These under researched biological effects of ion particles have a large impact on the health consequences of exposure. In this short review, we will discuss the effects of charged particles on an important biological process of the vascular system, angiogenesis, which creates and maintains the vasculature and is highly important in tumor vasculogenesis.

## Physical properties

All radiation is harmful to the vascular system, studies on the effects of photons like gamma rays show a cytotoxic effect leading to a number of vascular pathologies [1-4]. The physical properties of ion particles makes them even more effective cytotoxic agents than photons and thus potentially more suitable for radiotherapy, yet more dangerous as a coincident radiation in space. The main physical difference that affects the biological response between these two types of radiation is the energy deposition patterns in cells and tissues.

Ion particles penetrate matter in a straight track structure, produce secondary irradiations, and deposit energy per unit of track length, which is defined as the Linear Energy Transfer (LET). It can be thought of as a measure of the average thickness of the track with respect to energy deposition. As the particle traverses matter it remains at a constant speed and energy deposition until it starts to slow down. Correspondingly, the LET increases to higher values until the particle eventually stops. Plotted over distance the absorbed energy produces a Bragg curve where the LET remains at a plateau until it increases with the highest value at a peak near the end of the track, the Bragg peak [5]. Almost no dose is delivered to the normal tissue beyond the peak. It is these physical properties that make ion particles more effective for radiotherapy. The energy deposition can be focused on the tumor and not on normal tissue, more effectively than photons like gamma rays [6-9].

Ionizing photons scatter when they penetrate tissue. The same dose will produce more meandering tracks. Consequently, dose deposition for photon beam radiotherapy is maximal near the entrance of the tissue (skin), followed by an exponential decrease with tissue depth. Radiation of tumors inside the body, result in large doses delivered at the point of entry and unnecessary irradiation of surrounding normal tissues.

## Linear energy transfer and relative biological effect

Ionizing photons have a low LET and charged particles have variable LET’s which can range up to much higher values. This has an influence on the biological effectiveness of the radiation [10,11]. In a given material, such as human tissue, the LET value depends on the kinetic energy (velocity) and in the case of charged particles, also the mass, which is determined by the elemental species of the ion. Larger particles cause greater ionizations and secondary radiations, and therefore have a higher LET. For example, Hydrogen ions (protons) have a low mass compared to Fe ions and the corresponding LET’s for these particles at the same energy are 0.2 keV/μm and 150 keV/μm respectively, hundreds of times higher for Fe ions. For the same dose, high LET particles deposit the same energy from fewer particles than low LET particles. Thus, a tip cell undergoing vasculogenesis exposed to a dose of 75 cGy is estimated to receive approximately 40–45 particle traversals by Fe ions compared to 25000 traversals by low LET protons [12]. For many endpoints the biological effectiveness increases as the LET increases.

High-energy protons have a low LET similar to that of ionizing photons. Furthermore, although the deposition of photons at the tissue level is more scattered than that of protons, at the nanometer scale, the track patterns are similar (reviewed in [13]) and therefore might be expected to have a similar biological effect. However, there are still differences in the way protons and photons deposit energy [14,15] and therefore, there is always a potential for a differential biological response. As more studies on these radiations are carried out, more differences in the biological responses are revealed, including profound differences in the angiogenic response.

## The space environment and radiotherapy

In addition to a high biological effectiveness, high LET particles are highly penetrative through matter such as spacecraft shielding. For these reasons, the potential harm of space radiation is of particular concern. The space environment contains a complex mix of charged ions, with abundant low LET protons and less frequent high LET charged particles [16]. The risk estimates for both cancer and circulatory disease have recently been made for space radiation induced mortality and morbidity, and could exceed 5% and 10% [17]. The greater relative biological effectiveness of high LET particles makes them major contributors to the total dose equivalent, with iron ions being the principal contributor [18,19]. Solar particle events consisting of relatively large doses of mixed low LET protons also contribute to the radiation encountered in space. For radiotherapy, photon (gamma ray) therapy is widespread whereas charged particle therapy is much less frequent. It is relatively new, although increasingly used treatment. Low LET charged particles are utilized at proton facilities, and higher LET charged particles are utilized by Carbon ion facilities.

## Biological effects

Life on earth has not normally been exposed to charged particles so the biological effects of this exotic radiation are less well known than the effects of photons. DNA damage is a good example of the variety of lesions caused by charged particles compared to ionizing photons. Charged particles are known to cause more complex DNA damage [20-23]. Furthermore, a higher LET leads to even more complex damage which is harder to repair, resulting in more chromosomal aberrations and a higher risk of cancer. Differences in DNA repair are also seen in human endothelial vein cells (HUVEC) and capillary tissue models. Studies on the kinetics of DNA repair revealed differences between photons, low LET charged particles, and high LET charged particles [24]. High LET charged particles cause more persistent DNA damage than lower LET radiations and there are also significant differences between photons and protons of a similar LET. Such differences can also be seen in the effects of these radiations on the structure of mature human vessel models. High LET Fe ions are at least 4 times more effective than low LET protons at breaking down vessel structure, and 8 times more effective than gamma rays [25].

## Low LET radiation and angigenesis

Although the relative biological effectiveness of low LET charged particles and photons are similar as measured by long-established endpoints such as cell killing, there are a growing number of responses that show a higher biological effectiveness of charged particles such as protons. These include endpoints at the molecular, cellular, and tissue levels and have recently been reviewed in detail [26]. Biological processes such as gene expression, cell survival, apoptosis, inflammation, and cell invasion and migration, all show differences between photons and low LET protons. For angiogenesis, the difference is striking. Emerging evidence, in fact, indicates opposite effects of photons and low LET charged particles. Photons such as gamma rays are well known to promote angiogenesis and increase metastasis [27-32] often by causing an increase in the expression of pro-angiogenic factors such as vascular endothelial growth factor (VEGF), Interleukin 6 (IL-6), Hypoxia inducible factor 1 alpha (HIF-1α) and basic fibroblast growth factor (bFGF), in the irradiated tissue. Studies on the effects of low LET charged particles are relatively few but show that these radiations, in contrast, inhibit angiogenesis.

Investigations from the Hlatky laboratory demonstrate that low-LET proton irradiation significantly down-regulates some of the same and other pro-angiogenic factors including VEGF, IL-6, IL-8 and HIF-1α in human and murine cancer cells, and in primary human endothelial and fibroblast cells *in vitro *[33]. In the same study, co-culture experiments demonstrated that endothelial cell proliferation, and invasion, were inhibited by culturing with irradiated cancer or fibroblast cells. This suggests that proton irradiation may, in addition to direct action, contribute to angiogenesis suppression through modulation of paracrine signals from targeted cells. Most of these effects were seen at a dose of 1 Gy, which is too low for the induction of apoptosis in human capillary tissue models as measured by Terminal deoxynucleotidyl transferase dUTP nick end labeling (TUNEL) assay [25].

At the tissue level, our own studies on human vessel models (vasculogenesis model) support the notion that low LET particles inhibit vessel formation. Low LET protons inhibited vasculogenesis by human endothelial vein cells and human brain microvascular cells at relatively low doses. 40 cGy was sufficient to cause 50% inhibition and 80 cGy was sufficient for a full effect. Furthermore, this response was not seen after exposure to gamma photons. A dose of gamma rays 15 times higher (12 Gy) was required to inhibit the development of vessels to the same degree [25]. Experiments in animal models also indicate that low LET protons inhibit angiogenesis. The effects of a low-energy proton beam (35 MeV) on the development of blood vessels *in vivo* in Zebrafish embryos, was investigated. It was found that proton radiation dose-dependently reduced blood vessel formation in tissues [34]. Clinical studies also indicate an inhibition of angiogenesis by protons, the incidence of iris neovascularization in tissue of patients with uveal melanomas who had been irradiated with protons was investigated. 8 of 11 cases had incidence of neovascularization in the part of the iris tissue that did not receive proton irradiation, while neovascularization was not detected in areas receiving proton irradiation [35].

Taken together these results demonstrate that unlike photons, which stimulate angiogenesis, low LET protons at relatively low doses are potent inhibitors. They represent a hazard in the space environment but may be an advantage in radiotherapy.

## High LET radiation and angiogenesis

Studies on the effect of high LET charged particles are scarce due to limited access to the few facilities with colliders that can produce them. Most studies using high LET particles (and many low LET studies) have been carried out at the NASA Space Radiation Laboratory (NSRL), at Brookhaven National Laboratory (BNL) in Upton, New York.

With a higher LET these charged particles would be expected to be even more potent than low LET protons. However, our studies using human vessel models showed this not to be the case. Although the Fe ions were much more effective than low LET protons at disrupting the structure of mature vessel cultures, they were only as equally efficient at inhibiting vasculogenesis. As for low LET protons, 40 cGy was sufficient to cause 50% inhibition and 80 cGy was sufficient for a full effect. In another study, the particles were more effective. Exposure to carbon ions at the energy used in radiotherapy, a lower dose (10 cGy) of radiation inhibits developing vessel models and cell migration using ECV304 cells, a spontaneously transformed human endothelial cell line [36]. In an animal study, the effects of high LET Fe ions on mouse hippocampal microvessels was examined, and it was found that a dose as low as 50 cGy resulted in a loss of endothelial cells 1 year after irradiation [37].

**Figure 1:**
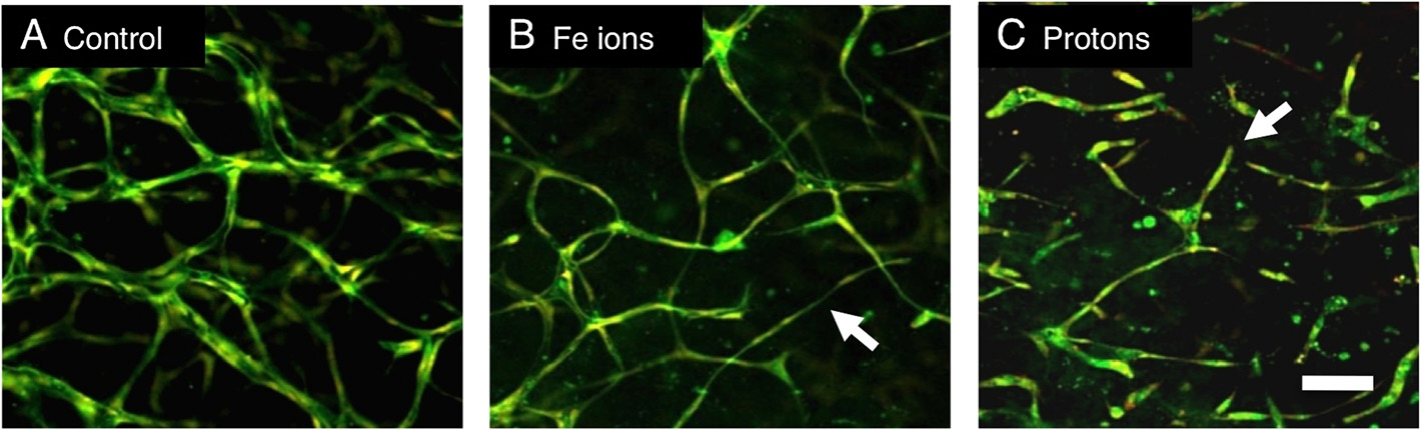
**Exposure to protons and Fe ions results in distinct morphologies of mature 3-Dimensional vessel models.** 24 hours after HUVEC were seeded into matrices they were exposed to 1Gy of each type of particle radiation and then cultured for a further 5 days until vessel structures had formed. Fixed cultures were stained for all protein material (DTAF – green) and nuclei (Propidium Iodide – red and imaging as yellow). Images are 10 slices 2 μm apart projected onto a single plane. **A**, Control HUVEC culture shows vessels with lumens that have formed a connecting network. **B**, Cultures exposed to 1 Gy Fe ions formed a network but vessels are often thinner without lumens (arrow). **C**, Cultures exposed to 1 Gy protons fail to form a network and vessels terminate in a dead end (arrow). Bar = 100 μm. From Grabham et al., 2013 [12].

Although the high LET particles clearly inhibit angiogenesis, they would be expected to be more effective than low LET particles if one follows the idea that higher LET leads to a more powerful biological effect, yet this is not the case. One possibility is that the low LET-mediated inhibition of angiogenesis is unusually potent because it is a separate type of inhibition. We have recently resolved this question by showing that low LET and high LET charged particles inhibit vasculogenesis by distinct mechanisms [12]. Clues came from differences in the final morphology of vessel cultures exposed to each type of radiation compared to the control cultures. Cells exposed to Fe ions extended narrow cellular processes and made connections to other cells but did not develop a central lumen whereas cells exposed to protons failed to make connections with other cells, cellular processes extended short distances into the gel matrix and terminated in a dead end (Figure 1). Further studies determined that each type of particle was inhibiting different stages of vessel growth. In the case of protons, the inhibition involves the regulation of PKC-dependent motile tips leading to a failure of cellular processes to migrate through the matrix, form guidance tunnels, and meet up with other cell processes. In the case of Fe ions, inhibition does not involve the blockage of motile tip activity since these structures are not affected. Instead, the cells fail to form widened tunnels in the matrix and lumen-containing tubular structures at the later stages of vasculogenesis.

The effect of high LET charged particles is distinct from that of low LET protons, there are two separate mechanisms whereby charged particles inhibit vessel growth according to the physical properties of the particle.

## Conclusions

In summary, the effects of ionizing radiations on angiogenesis are more complex than might be expected. There are now at least three distinct ways in which radiation can affect vessel growth. 1) The photons of electromagnetic radiations stimulate vessel growth at least in part, by causing the increased expression of angiogenic factors. 2) Low LET charged particles like protons inhibit angiogenesis by an unknown mechanism although decreased expression of angiogenic factors and reduced motile tip activity is implicated. 3) High LET heavy ions like Fe ions also inhibit angiogenesis by an unknown mechanism that affects the later stages of tubulogenesis. This complexity of response opens up possibilities of greater control over angiogenesis and the resulting pathologies during coincident exposure or therapy. For exposure in space, knowledge of these mechanisms will enable more precise risk assessment and mitigation strategies. For radiotherapy, treatment could be manipulated to utilize the radiation effectively. In addition, effectiveness can be increased further when used in the right combination of anti-angiogenic drugs. Further research in this field should contribute to a great improvement in these strategies.

## Competing interests

The authors declare that they have no competing interests.

## Authors’ contributions

PG. Prepared and wrote the manuscript. PS. Prepared and wrote the manuscript. Both authors read and approved the final manuscript.

## References

[B1] Graebe A Schuck EL Lensing P Putcha L Derendorf H Physiological, pharmacokinetic, and pharmacodynamic changes in space J Clinical Pharmacol 2004 5 837 853 10.1177/0091270004267193 15286087

[B2] Jurado JA Bashir R Burket MW Radiation-induced peripheral artery disease Catheterization Cardiov Interv: Off J Soc Cardiac Angiogr Interv 2008 5 563 568 10.1002/ccd.21681 18819153

[B3] Little MP Azizova TV Bazyka D Bouffler SD Cardis E Chekin S Chumak VV Cucinotta FA de Vathaire F Hall P Systematic review and meta-analysis of circulatory disease from exposure to low-level ionizing radiation and estimates of potential population mortality risks Environ Health Perspectives 2012 5 1503 1511 10.1289/ehp.1204982 PMC3556625 22728254

[B4] Preston DL Shimizu Y Pierce DA Suyama A Mabuchi K Studies of mortality of atomic bomb survivors. Report 13: solid cancer and noncancer disease mortality: 1950–1997 Radiation Res 2003 5 381 407 10.1667/RR3049 12968934

[B5] Wilson RR Radiological use of fast protons Radiology 1946 5 487 491 20274616 10.1148/47.5.487

[B6] Durante M Loeffler JS Charged particles in radiation oncology Nat Rev Clinical Oncol 2010 5 37 43 10.1038/nrclinonc.2009.183 19949433

[B7] Jakel O Medical physics aspects of particle therapy Radiat Prot Dosimetry 2009 5 156 166 10.1093/rpd/ncp192 19828718

[B8] Jones B The case for particle therapy British J Radiol 2006 5 24 31 10.1259/bjr/81790390 16421401

[B9] Jones B The potential clinical advantages of charged particle radiotherapy using protons or light ions Clinical Oncol 2008 5 555 563 10.1016/j.clon.2008.02.012 18462929

[B10] Nelson GA Fundamental space radiobiology Gravitation Space Biol Bull: Publ Am Soc Gravitation Space Biol 2003 5 29 36 12959129

[B11] Schimmerling W Cucinotta FA Dose and dose rate effectiveness of space radiation Radiation Prot Dosimetry 2006 5 349 353 10.1093/rpd/ncl464 17169950

[B12] Grabham P Sharma P Bigelow A Geard C Two distinct types of the inhibition of vasculogenesis by different species of charged particles Vascular Cell 2013 5 16 10.1186/2045-824X-5-16 24044765 PMC3856512

[B13] Goodhead DT Energy deposition stochastics and track structure: what about the target? Radiation Prot Dosimetry 2006 5 3 15 10.1093/rpd/ncl498 17276998

[B14] Dicello JF Absorption characteristics of protons and photons in tissue Technol Cancer Res Treat 2007 5 25 29 17668948 10.1177/15330346070060S404

[B15] Liamsuwan T Uehara S Emfietzoglou D Nikjoo H Physical and biophysical properties of proton tracks of energies 1 keV to 300 MeV in water Int J Radiation Biol 2011 5 141 160 10.3109/09553002.2010.518204 21281230

[B16] Zeitlin C Hassler DM Cucinotta FA Ehresmann B Wimmer-Schweingruber RF Brinza DE Kang S Weigle G Bottcher S Bohm E Measurements of energetic particle radiation in transit to mars on the mars science laboratory Science 2013 5 1080 1084 10.1126/science.1235989 23723233

[B17] Cucinotta FA Kim M-HY Chappell LJ Huff JL How safe is safe enough? Radiation risk for a human mission to mars PloS One 2013 5 e74988 10.1371/journal.pone.0074988 24146746 PMC3797711

[B18] Durante M Kronenberg A Ground-based research with heavy ions for space radiation protection Adv Space Res: Off J Committee Space Res 2005 5 180 184 10.1016/j.asr.2004.12.034 15934192

[B19] Held KD Effects of low fluences of radiations found in space on cellular systems Int J radiation Biol 2009 5 379 390 10.1080/09553000902838558 19382021

[B20] Asaithamby A Chen DJ Mechanism of cluster DNA damage repair in response to high-atomic number and energy particles radiation Mutation Res 2011 5 87 99 10.1016/j.mrfmmm.2010.11.002 21126526 PMC3318975

[B21] Blakely EA Kronenberg A Heavy-ion radiobiology: new approaches to delineate mechanisms underlying enhanced biological effectiveness Radiation Res 1998 5 S126 S145 10.2307/3579815 9806616

[B22] Durante M Cucinotta FA Heavy ion carcinogenesis and human space exploration Nature Rev Cancer 2008 5 465 472 10.1038/nrc2391 18451812

[B23] Prise KM Pinto M Newman HC Michael BD A review of studies of ionizing radiation-induced double-strand break clustering Radiation Res 2001 5 572 576 10.1667/0033-7587(2001)156[0572:AROSOI]2.0.CO;2 11604074

[B24] Grabham P Bigelow A Geard C DNA damage foci formation and decline in two-dimensional monolayers and in three-dimensional human vessel models: differential effects according to radiation quality Int J Radiation Biol 2012 5 493 500 10.3109/09553002.2012.679382 22449005

[B25] Grabham P Hu B Sharma P Geard C Effects of ionizing radiation on three-dimensional human vessel models: differential effects according to radiation quality and cellular development Radiation Res 2011 5 21 28 10.1667/RR2289.1 21175343

[B26] Girdhani S Sachs R Hlatky L Biological effects of proton radiation: what we know and don’t know Radiation Res 2013 5 257 272 10.1667/RR2839.1 23373900

[B27] Moeller BJ Cao Y Li CY Dewhirst MW Radiation activates HIF-1 to regulate vascular radiosensitivity in tumors: role of reoxygenation, free radicals, and stress granules Cancer Cell 2004 5 429 441 10.1016/S1535-6108(04)00115-1 15144951

[B28] Park CM Park MJ Kwak HJ Lee HC Kim MS Lee SH Park IC Rhee CH Hong SI Ionizing radiation enhances matrix metalloproteinase-2 secretion and invasion of glioma cells through Src/epidermal growth factor receptor-mediated p38/Akt and phosphatidylinositol 3-kinase/Akt signaling pathways Cancer Res 2006 5 8511 8519 10.1158/0008-5472.CAN-05-4340 16951163

[B29] Sofia Vala I Martins LR Imaizumi N Nunes RJ Rino J Kuonen F Carvalho LM Ruegg C Grillo IM Barata JT Low doses of ionizing radiation promote tumor growth and metastasis by enhancing angiogenesis PloS One 2010 5 e11222 10.1371/journal.pone.0011222 20574535 PMC2888592

[B30] Sonveaux P Brouet A Havaux X Gregoire V Dessy C Balligand JL Feron O Irradiation-induced angiogenesis through the up-regulation of the nitric oxide pathway: implications for tumor radiotherapy Cancer Res 2003 5 1012 1019 12615716

[B31] Gorski DH Beckett MA Jaskowiak NT Calvin DP Mauceri HJ Salloum RM Seetharam S Koons A Hari DM Kufe DW Weichselbaum RR Blockage of the vascular endothelial growth factor stress response increases the antitumor effects of ionizing radiation Cancer Res 1999 5 3374 3378 10416597

[B32] Hlatky L Tsionou C Hahnfeldt P Coleman CN Mammary fibroblasts may influence breast tumor angiogenesis via hypoxia-induced vascular endothelial growth factor up-regulation and protein expression Cancer Res 1994 5 6083 6086 7525053

[B33] Girdhani S Lamont C Hahnfeldt P Abdollahi A Hlatky L Proton irradiation suppresses angiogenic genes and impairs cell invasion and tumor growth Radiation Res 2012 5 33 45 10.1667/RR2724.1 22702646

[B34] Jang GH Ha JH Huh TL Lee YM Effect of proton beam on blood vessel formation in early developing zebrafish (Danio rerio) embryos Archives Pharmacal Res 2008 5 779 785 10.1007/s12272-001-1226-1 18563361

[B35] Boyd SR Gittos A Richter M Hungerford JL Errington RD Cree IA Proton beam therapy and iris neovascularisation in uveal melanoma Eye 2006 5 832 836 10.1038/sj.eye.6702072 16167079

[B36] Takahashi Y Teshima T Kawaguchi N Hamada Y Mori S Madachi A Ikeda S Mizuno H Ogata T Nojima K Heavy ion irradiation inhibits *in vitro* angiogenesis even at sublethal dose Cancer Res 2003 5 4253 4257 12874034

[B37] Mao XW Favre CJ Fike JR Kubinova L Anderson E Campbell-Beachler M Jones T Smith A Rightnar S Nelson GA High-LET radiation-induced response of microvessels in the hippocampus Radiation Res 2010 5 486 493 10.1667/RR1728.1 20334521

